# Calibration of force detection for arbitrarily shaped particles in optical tweezers

**DOI:** 10.1038/s41598-018-28876-y

**Published:** 2018-07-17

**Authors:** Ann A. M. Bui, Anatolii V. Kashchuk, Marie Anne Balanant, Timo A. Nieminen, Halina Rubinsztein-Dunlop, Alexander B. Stilgoe

**Affiliations:** 1 School of Mathematics and Physics, The University of Queensland, St. Lucia, QLD 4072 Australia; 2 Australian Red Cross Blood Service, Brisbane, QLD 4059 Australia; 3 Science and Engineering Faculty, Queensland University of Technology, Brisbane, QLD 4000 Australia

## Abstract

Force measurement with an optical trap requires calibration of it. With a suitable detector, such as a position-sensitive detector (PSD), it is possible to calibrate the detector so that the force can be measured for arbitrary particles and arbitrary beams without further calibration; such a calibration can be called an “absolute calibration”. Here, we present a simple method for the absolute calibration of a PSD. Very often, paired position and force measurements are required, and even if synchronous measurements are possible with the position and force detectors used, knowledge of the force–position curve for the particle in the trap can be highly beneficial. Therefore, we experimentally demonstrate methods for determining the force–position curve with and without synchronous force and position measurements, beyond the Hookean (linear) region of the trap. Unlike the absolute calibration of the force and position detectors, the force–position curve depends on the particle and the trapping beam, and needs to be determined in each individual case. We demonstrate the robustness of our absolute calibration by measuring optical forces on microspheres as commonly trapped in optical tweezers, and other particles such a birefringent vaterite microspheres, red blood cells, and a deformable “blob”.

## Introduction

Calibration for force measurement is often performed per-particle, using methods that assume that the trap is Hookean, i.e., can be characterised by a spring constant, and depend on knowing the viscous drag acting on the particle, and thus depend on knowing the particle size and shape, and the viscosity (and thus temperature) of the surrounding medium. Two common methods are finding the spring constant from the corner frequency of the power spectrum^1–5^ and using viscous drag to move the particle away from the equilibrium position and thus determine the spring constant^6^. While these methods have been very useful in cases where the particle and surrounding environment are well-known, the assumptions required by these methods make them difficult to use for unknown particles in poorly-known environments. One solution is to use absolute calibration of the force detection system.

The concept of absolute calibration of a force detector is not new. The transverse optical gradient force arises from deflection of the transmitted beam by the particle. This introduces a transverse momentum flux into the beam, which, by conservation of momentum, results in a transverse optical force acting on the particle^7^. If the deflection of the beam can be measured, for example, using a CCD camera as we suggested in 2001^8^, the optical force can be measured. If a CCD camera is used, the transverse momentum flux can be determined from the image. This is equivalent to determining the shift of the “centre of mass” of the image (i.e., the intensity-weighted average position).

A position-sensitive detector (PSD) which directly gives the position of the centre of mass of the beam provides a convenient alternative detector, with potential advantages in data capture rate, signal-to-noise, and dynamic range over a CCD camera. While a quadrant photodiode (QPD) can be used to measure the deflection of a laser beam, a QPD is unsuitable for absolute calibration since the signal corresponding to a particular deflection of the beam depends on the width of the beam at the QPD. Since the width of the beam depends on the divergence of the transmitted beam, which in turn depends on the axial optical force, the width will change from particle to particle. In addition, if the trapping beam is changed, the beam width at the QPD can change. Further, for some types of trapping beam beyond the fundamental Gaussian mode, such as Hermite–Gauss modes or similar, the signal from the QPD can fail to be linear with deflection, even for small deflections of the beam. A PSD, on the other hand, can provide a consistent signal even when the beam width at the PSD changes, and so can be used for beams such as HG_10_ beams.

There has been significant previous experimental work in this area. For example, Smith *et al*.^9^ used measured properties of the optical system delivering the transmitted beams to a pair of PSDs for a counter-propagating trap to determine an absolute calibration for force measurement. A similar method was used by Farré and Montes-Usategui^10^ for a single-beam trap; this required improvements to deal with the collection of the transmitted light by a high numerical aperture objective. More recently^11^, this has been extended to rod-shaped particles. Another approach is to use back-focal-plane interferometry to measure the deflection of the transmitted trapping beam^12^. In our current paper, we add to this body of work by, first, demonstrating a simple method for the positioning and calibration of a PSD for force measurement through deflection of the trapping beam, which requires relatively little knowledge of the optical system, and second, enhancing the capabilities of an optical trapping system with such absolute force detection by using the force–position relationship of a trapped particle.

For some purposes, optical force measurements are sufficient. However, in many cases, pairs of force and position measurements are needed. For example, if one is measuring the mechanical properties of DNA, the desired data are typically the dependence of the tension of the DNA strand with its extension^1,6^. To determine the position, one can use a CCD camera. This is amenable to absolute calibration, since the distance in the image plane per pixel on the CCD can be readily found.

It is also possible to use the relationship between the optical force and the position of the particle within the trap. For a spherical particle, the optical force is a function of its position in the trap. If this function has been measured or calculated a priori^13^, the force corresponding to a given position is known. Therefore, for regions within the trap where the force–position function is one-to-one (and so, the position is a function of the force, as well as the force being a function of the position), the position corresponding to a given force is known. For a non-spherical particle, the force can also depend on the alignment. If the alignment is sufficiently close to constant (e.g., the surroundings hold the particle in a fixed orientation, or the trap holds the particle in a fixed orientation), the force–position relationship can be treated as a function of position, for a given alignment. More generally, it is often possible to treat the force–position relationship as a function of position in a time-averaged sense^14^.

Therefore, to obtain paired force and position measurements, one can either (a) obtain simultaneous measurements using the camera and PSD, or (b) first determine the force–position relationship for the particle in the trap, and then measure either the force or the position, and determine the other from the force–position curve. The latter method, using the force–position relationship has the disadvantage of the force–position relationship needing to be found first. This is an additional calibration procedure that needs to be performed for each particle in each trap, and cannot be dealt with as a once-only calibration such as the absolute calibration of the force detector (PSD). However, there can be compensating benefits that make this method highly advantageous. First, it can be technically difficult to obtain sufficiently simultaneous position and force measurements from the camera and PSD. Typically, *exactly* simultaneous is not possible—the question is whether position and force measurements sufficiently close together in time to be considered simultaneous in practice are possible. This depends on details of the instruments and the data acquisition system. Second, even if effectively simultaneous measurements are possible, such measurement can proceed no faster than the slower of the two instruments. For example, the PSD used to measure the optical force might be capable of making measurements at a rate of tens of kHz, while the camera used to measure the position might be limited to a maximum frame rate of kHz. Measurement using the force–position relationship can obtain data at the speed of the faster of the two instruments. Third, knowledge of the force–position curve can be used to detect changes in the trap, such as the migration of an additional particle into the trap. Therefore, we will discuss and demonstrate methods for determining the force–position relationship for optically trapped particles using an absolutely-calibrated force detection system.

Many methods of determining the force–position relationship only yield a small portion of the force–position curve. For example, the force–position relationship is often given by the spring constant for the trap, so only the Hookean (linear) region near the equilibrium position is described. The very commonly used power spectrum method^1–5^ not only assumes that the trap is Hookean, but also depends on knowledge of the viscous drag, which assumes that the viscosity of the trapping medium, size and shape of the trapped particle are known. Some methods which do not involve finding a spring constant, such as displacing the particle from the equilibrium position using viscous drag (e.g., by moving the stage) also require knowledge of the viscous drag^15,16^. Such drag-based methods become difficult when the force measurement is to be done in an unknown environment with an uncharacterised probe particle. For example, if a force measurement was to be done inside a cell, with a cell organelle being used as the probe particle, properties such as the viscosity and the size and shape of the particle would be unknown, as would the effect of the cellular membrane on the measurement. Therefore, it is important to consider methods where the least amount of external information is required^4,14,17,18^.

The main group of methods that are independent of viscous drag that are used to obtain information about the force–position relationship make use of the fact that the position distribution of the particle in the trap is given by the Boltzmann distribution. These methods require position measurements, and knowledge of the absolute temperature of the optical trap. While the temperature is not always known exactly, e.g., if there is unknown heating in the trap due to the trapping beam, error due to temperature differing from the assumed temperature is usually small (as we will show below), unlike the often large error due to temperature effects on viscosity in drag-dependent methods. Calibration through Boltzmann statistics can be done in two ways: with or without assuming a Hookean trap shape. If the trap is Hookean, the position distribution will be Gaussian, and this assumption can be used to fit a Gaussian distribution to the position measurements, greatly reducing the statistical error. We will refer to this method, assuming a Hookean (linear) trap, as the *equipartition method*. If the assumption of a Hookean trap is not made, the potential, and hence the force, can be calculated from particle position distribution directly^14^. Hereon in, we will refer to this method as the *Boltzmann statistics method*. This method requires discretising the position measurements, and the accuracy improves with increased resolution (i.e., spatially smaller bins into which the position measurements are discretised), but only until the error from numerically differentiating the potential to obtain a force becomes large. This leaves an optimal resolution when using Boltzmann statistics. The particle will have a lower probability to be at the edges of the trap, resulting in poor edge statistics. Both the Boltzmann statistics and equipartition force calibration methods make use of Brownian motion to move the particle to traverse the trapped region, and hence only a small region near the equilibrium position is explored for a typical optical trap.

While the Boltzmann statistics method can be applied to arbitrary particles in arbitrary traps, and can be used in a time-averaged sense^14^ for non-spherical particles that change orientation, these three drawbacks—limited resolution, limited range of motion, and poor edge statistics—mean that improvements are highly desirable. Below, we will demonstrate with both computational simulation of calibration measurements and with experimental results that such improvements are possible using a force detector with absolute calibration. The Boltzmann statistics method will also be used as a baseline comparison for methods using absolute force measurement to obtain the force–position relationship.

Here, in addition to absolute calibration of a force detector (in our setup, a PSD), we demonstrate two methods to obtain the force–position relationship. Neither depends on knowledge of the viscous drag, or even of the temperature. Both methods can be applied to arbitrary and even unknown particles.

First, if synchronous force and position measurements are possible, a series of paired force and position measurements will give the force–position relationship. Brownian motion can be used to provide the range of positions. If the force–position relationship is desired over a larger spatial region than is explored through Brownian motion, the particle can be displaced further from the equilibrium position by fluid flow, e.g., by moving the stage uni-directionally or by oscillating the stage. It is important to note that while viscous drag is used to move the particle away from the optical equilibrium position, it is not necessary to know the viscous drag force, since the absolutely calibrated detector provides the required force information. We refer to this method below as the *synchronous method*.

Second, if synchronous force and position measurements are not possible, unpaired force and position data can be collected. Since these data are not paired, the force and position measuring instruments do not need to operate at the same data acquisition rates, and the size of the force and position data sets need not be identical. They should be collected over the same time period, or at least over time periods over which the statistics of the particle behaviour are the same. This method requires us to assume that the force–position curve is monotonic. With this assumption, it follows that if the position measurements are sorted, and the force measurements are sorted, then they should correspond to each other, as shown in the supplementary information (Section 1). The force–position relationship can then be obtained as the quantile–quantile plot (*qq*-plot) of the force and position data sets. We refer to this method below as the *mapping method*.

Below, we will first describe our simple procedure for absolute calibration of a PSD. Next, we will use simulated measurements to compare the synchronous and mapping methods for determining the force–position relationship. This will be followed by experimental demonstration of these methods, leading to absolute force measurement for particles including non-spherical particles, birefringent particles, and deformable particles, as well as when using non-Gaussian beams.

## Absolute calibration of force detector

**Figure 1:**
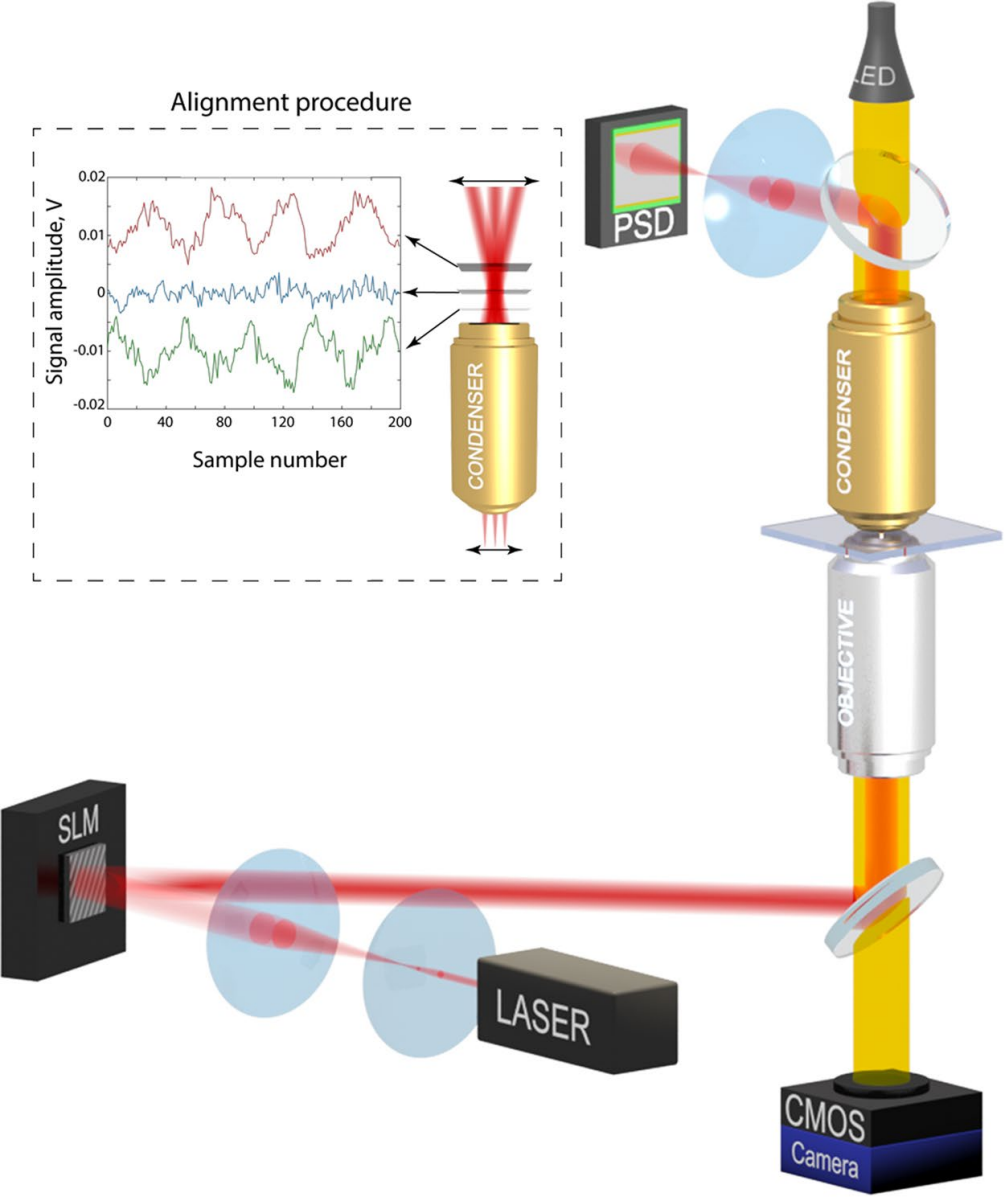
Optical setup for force measurements. Dichroic mirrors (DM) are used to separate the trapping light and illumination. The inset shows the PSD alignment procedure using SLM. Curves show optimal, under- and overshot positions (signal amplitudes are shifted for better visibility).

We extend on the experimental setup of References^10,12,19^, by modifying the calibration of the PSD. The apparatus used is shown in Fig. 1. We calculate a calibration constant from measurements of a trapped particle, instead of using geometrical measurements of the optical system. This simplifies calculation of the calibration constant if optical paths of the detection system has changed, e.g. the change of the focal distance of the condenser due to a heating from high-power trapping laser or intentional heating of the condenser in order to comply with biological experiments requirements.

To detect the angular distribution of light the PSD is placed in the Fourier plane of the condenser. This plane can be imaged by the relay lens (1:1 magnification with 2F object-lens and lens-image distance). By using the property of the image formation we shift the trapping beam in the object’s plane (inset in Fig. 1). The correct position of the detector can be identify by the smallest beam displacement which can be achieved by monitoring the PSD output voltages.

A spatial light modulator (SLM) is programmed to continuously oscillate the beam. This results in a triangular profile of the beam position available from the PSD. By changing the distance between the PSD and relay lens we are looking for the position with the smallest measured displacement (inset in Fig. 1).

Light scattered by the particle is collected by a condenser with a high numerical aperture to collect as much light as possible including rays scattered at large angles. The back focal plane of the condenser is imaged onto the PSD to obtain the optical force. A CMOS camera is used to track the position of the particle. The PSD must be calibrated with a trapped particle using position measurements from the camera. This calibration is absolute due to the nature of the method and will work for a wide variety of particles and environments. The calibration coefficient needs only to be updated when we change the optical paths or components. The calibration coefficient does not need to be changed if the particle is changed, or the trapping medium is changed, or even if the shape of the trapping beam is changed. We use the equipartition theorem to calibrate the PSD. The position distribution of a particle in the trap will follow the Boltzmann distribution:

1 $$P=\exp (\,{-}^{U(x)}\hspace{-1pt}\,{/}_{{k}_{B}T}),$$

where *k*_*B*_ is Boltzmann’s constant, *T* is the absolute temperature, *U*(*x*) is the trapping potential, and *x* is the position of the particle. For calibration we assume that trap is linear, with the force being *F* = −*kx*, where *k* is the trap stiffness. The trapping beam, trapping power, and trapped particle can be chosen to make this an accurate assumption; after calibration these restrictions are not needed. For a linear trap,

2 $$U(x)=\frac{1}{2}k{x}^{2},$$

and the particle position distribution is

3 $$P=\exp (-\frac{k{x}^{2}}{2{k}_{B}T}).$$

This is a normal (Gaussian) distribution described by

4 $$P=\exp (-\frac{{x}^{2}}{2{\sigma }^{2}}).$$

Here we assume that the mean position *x* is zero. The standard deviation is given by

5 $$\sigma =\sqrt{\frac{{k}_{B}T}{k}}$$

Using the camera, we measure the position distribution of the trapped particle. From the PSD, we have corresponding data, still uncalibrated, measured as voltages. We need to convert this data into units of force (newtons), such that

6 $${F}_{{\rm{N}}}={C}_{{\rm{N}}/{\rm{V}}}{F}_{V},$$

where *C*_N/V_ is a calibration constant, and the subscripts indicate the units (V for voltage in volts, N for force in newtons, and m for position in metres). Thus,

7 $${F}_{{\rm{V}}}=-\,{k}_{{\rm{V}}/{\rm{m}}}{x}_{m},$$

8 $${F}_{{\rm{N}}}=-\,{C}_{{\rm{N}}/{\rm{V}}}{k}_{{\rm{V}}/{\rm{m}}}{x}_{m},$$

9 $${k}_{{\rm{N}}/{\rm{m}}}={C}_{{\rm{N}}/{\rm{V}}}{k}_{{\rm{V}}/{\rm{m}}},$$

and

10 $${k}_{{\rm{V}}/{\rm{m}}}=\frac{{k}_{{\rm{N}}/{\rm{m}}}}{{C}_{{\rm{N}}/{\rm{V}}}}.$$

From the position distribution, we can obtain the optical force distribution

11 $$P=\exp (\frac{-k{x}^{2}}{2{k}_{B}T})=\exp (\frac{-{k}_{{\rm{N}}/{\rm{m}}}{{F}_{V}}^{2}}{2{k}_{{\rm{V}}/{\rm{m}}}^{2}{k}_{B}T})$$

Using equation (10),

12 $$P=\exp (\frac{-{C}_{{\rm{N}}/{\rm{V}}}^{2}{{F}_{V}}^{2}}{2{k}_{{\rm{N}}/{\rm{m}}}{k}_{B}T}),$$

and the standard deviation of the optical force distribution measured in volts is

13 $${\sigma }_{f}=\sqrt{\frac{{k}_{{\rm{N}}/{\rm{m}}}{k}_{B}T}{{C}_{{\rm{N}}/{\rm{V}}}^{2}}}\,.$$

Now, we can substitute the spring constant from the camera position data into the force standard deviation:

14 $${\sigma }_{{\rm{cam}}}=\sqrt{\frac{{k}_{B}T}{{k}_{{\rm{N}}/{\rm{m}}}}}\to {k}_{{\rm{N}}/{\rm{m}}}=\frac{{k}_{B}T}{{\sigma }_{{\rm{cam}}}^{2}}$$

15 $${\sigma }_{f}=\frac{{k}_{B}T}{{C}_{{\rm{N}}/{\rm{V}}}{\sigma }_{{\rm{cam}}}},$$

where *σ*_cam_ is the standard deviation of the position (measured in meters). Finally, the PSD force calibration coefficient, needed to convert the measured voltage signal to a force in newtons, is

16 $${C}_{{\rm{N}}/{\rm{V}}}=\frac{{k}_{B}T}{{\sigma }_{f}{\sigma }_{{\rm{cam}}}},$$

or, if we know the trap stiffness,

17 $${C}_{{\rm{N}}/{\rm{V}}}={k}_{{\rm{N}}/{\rm{m}}}\frac{{\sigma }_{{\rm{cam}}}}{{\sigma }_{f}}.$$

It should be noted that detectors for both position and force need to be faster than the relaxation time of the particle in the trap to correctly reflect its statistics. Similar calibration procedures have been used for QPDs^20^ (in which case, they do not yield an absolute calibration, as noted above). While this calibration is performed using a particular particle, in the linear region of the trap, the calibration coefficient *C*_N/V_ can be used for arbitrary particles, and outside the linear region of the trap.

## Results

### Computational simulation

It is important to evaluate the accuracy of different calibration methodologies as each will be influenced by particular experimental conditions such as the local environment in the sample or the optical system. We test and compare several different methods of determining the force–position curve, each subject to a different constraint in a proposed experiment. We evaluate force–position curves in the cases of: synchronised measurements of force and position, force and position measurements without synchronisation, and position-only measurements using a camera (i.e., without force measurements).

Unlike an experiment, an accurate optical tweezers simulation will automatically yield optical forces, as these are required for the simulation. We will, however, take account of this ground truth through the addition of noise of varying kinds to the simulation where necessary. This will allow us to compare the absolute error of each method to that of just sampling the force through the Boltzmann statistics of thermal excitations. Our definition of error in these methods is given in the supplementary information (Section 2).

An optical tweezers is often simulated as a particle subject to a Hookean restoring force. This approach can be quite accurate as long as the particle remains extremely close to its equilibrium position. However, real optical traps often present measurably non-linear restoring forces, and it is useful to performance simulations with realistic optical forces.

**Figure 2:**
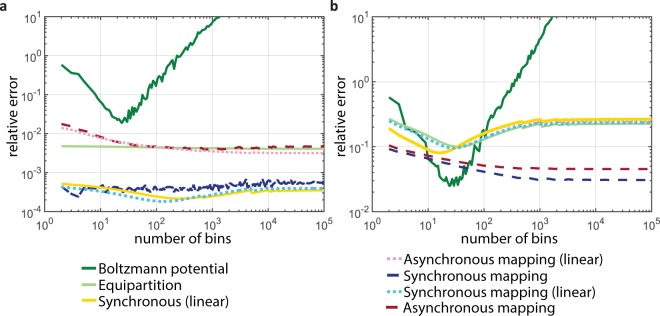
A comparison of the relative error of calibration against actual stiffness over spatial binning resolution for different calibration methods for (**a**) An optical trap simulated with the OTT and (**b**) modified cubic Hookean spring model of an optical trap. The dark green lines correspond to the relative error derived from the Boltzmann statistics calibration method. The light green lines are from equipartition analysis. The yellow curves are the error from finding the ratio between averages of synchronised position and force data. The dashed dark red curves represent the error from the asynchronous quantile mapping calibration. The dashed pink curves represent the error of the line of best fit to the quantile-quantile curve for asynchronous mapping. The dark blue curves show the error when capturing simultaneous force–position data using the quantile mapping calibration. The light blue curves represent the error for the line of best fit to the synchronious force and position measurements.

First, we perform simulations using the Optical Tweezers Toolbox^21^ (OTT) to compute optical forces acting on a dielectric sphere under the influence of the thermal bath. This will yield an accurate model of optical tweezers under the influence of strong forces moving the particle far from unperturbed equilibrium much like that which occur in experiments involving the extension of a linked molecule or high velocity drag measurements. The position of the particle in our simulation is updated through a Runge–Kutta simulation of the resulting Langevin equation^14,22^. The simulated force and position measurements were extracted from the simulation to match the data capture rate achieved in the experiments. The results of this computational investigation are summarised graphically Fig. 2. Figure 2a depicts the relative error for various calibration methods using optical force data from the OTT.

Second, in order to explore the behaviour of these methods in a more strongly non-linear trap, we perform simulations were we assume that the restoring force is cubic, with a linear Hookean term and a cubic term. Figure 2b shows the relative error for the same calibration methods under the cubic restoring force. These simulations add 1% noise to the force and position measurements.

The Boltzmann statistics can be used to find the optical potential through sampling the occupation probability of a particular position microstate over a long period of time. However, there is a trade off between the number statistics and the spatial resolution needed to satisfy the approximation that the trap has uniform properties over a single bin. The effect of this is shown in both Fig. 2a and b, where a minimum uncertainty occurs due to the change in trap stiffness (curvature). Our simulation results show that there is an optimal bin size using Boltzman statistics.

Consider a sequence of force and position data in time. Often in an experiment, force and position measurements are not captured in an ideal synchronous manner, due to line delays, capture from different un-triggered apparatus, etc. We can accounted for these scenarios in our analysis by treating the force and position data as a set of temporally uncorrelated points; we have labelled calibrations from treatment of data in this way as non-(or a)synchronous. For both types of simulation, the synchronous and asynchronous quantile mapping methods have similar levels of error, which can be seen in Fig. 2 (dark red/blue curves). We see that in general, the force–position relationships obtained using synchronous measurements are more accurate than those obtained from analysis of non-synchronous data. We can perform the same sets of analysis using our trap models assuming that the traps were Hookean (as is common). This relative errors from this analysis is shown with the dashed pink and light blue curves. The direct quantile–quantile mapping calibrations generally have smaller relative differences between their predicted stiffness and the actual trap stiffness than the stiffness predicted from analysis assuming a linear trap response.

A linear fit to the synchronous pairs of force and position data (for the synchronous cases) and the matched quantiles (for the asynchronous cases) allows the effect of noise to be reduced by averaging. However, it introduces additional error due to the trap not being linear. For the “real” trap (Fig. 2a), the force–position curve is close to linear, and a modest improvement in the error results from such a linear fit. For the cubic trap, where the nonlinear component is larger than in the “real” trap, the linear fit results in increased error. In general, if known, the appropriate trap shape would need to be assumed for a calibration from a curve fit of synchronous force and position measurements.

As with all calibrations of experimental arrangements, there will always be noise in the system. Our experimental setup has an associated noise of about 10% which does not greatly affect the calibration. A further discussion of noise in the system is given in the Supplementary information (Sections 3 and 4).

**Figure 3:**
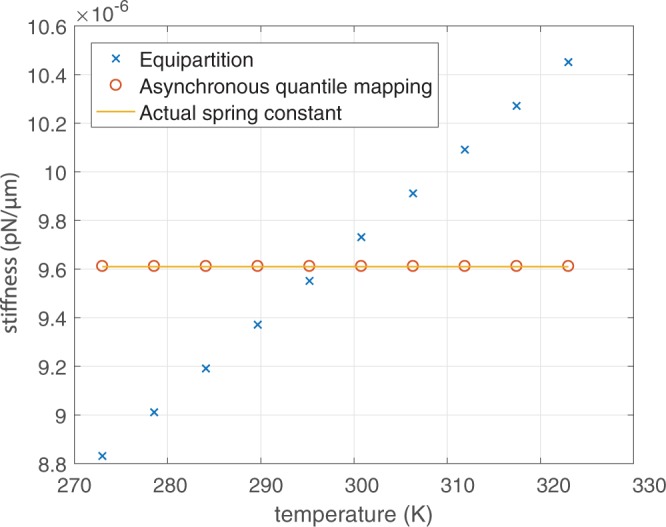
Comparison of the spring constant from the equipartition method and from the mapping method with the actual spring constant of the trap.

If we assume that an accurate calibration of the force detector can be performed, then future force measurements are independent of the temperature. We can demonstrate that an accurate force–position relationship can be found accurately regardless of any heating of the trap through absorption of the trapping beam. Figure 3 shows how the spring constant (for an assumed-Hookean trap) is affected by temperature using methods such as Boltzmann statistics, and is unaffected when using our quantile–quantile mapping method.

Since our original calibration of the force detector makes use of the equipartition theorem (Eq. (16)), it is important to avoid heating when performing this calibration. Heating can be avoided by using a low trapping power, and a particle and surrounding medium with low optical absorption, for this initial calibration.

### Experimental measurements

**Figure 4:**
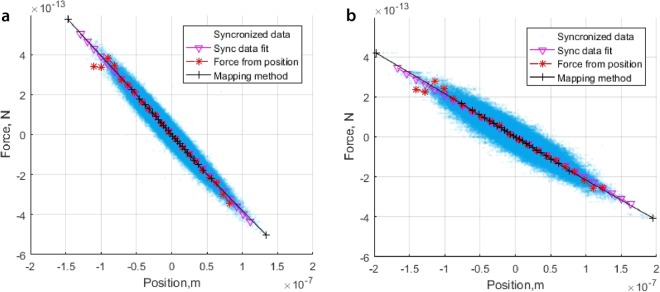
Force calculation methods comparison for two particles of different sizes: (**a**) 1.9 *μm* and (**b**) 4.9 *μm*. The comparison of the force curves calculated from the position distribution, using the Boltzmann statistics method, using the mapping method and by fitting the synchronized data.

We trap particles of different sizes, materials, shapes in a various mediums and measure the force acting on each particle using the calibrated PSD. In Fig. 4a,b, the synchronised calibration results are compared with the force obtained from position measurements using the equipartition theorem and building a potential, and with the proposed mapping method. The averaged difference in the trap stiffness for these cases is less than 2% (see Table 1). Some deviations from the theoretical straight line are explained by the poor statistics at the edges of the position distribution. A series of measurements of the calibration constant with different samples (silica and polystyrene particles) and on different days (10 measurements) show a good performance. The standard error over all measurements is $CSE=1.40\cdot {10}^{-14}$ N/V which is $\approx$1% of the mean value ${C}_{N/V}=1.50\cdot {10}^{-12}$ N/V. Of course, the accuracy of these measurements will still be limited by the uncertainty in the temperature when the calibration is performed, and the uncertainty in the camera calibration. Further, noise in the system (approximately 10% in our case, as discussed in the Supplementary material (Section 4)) will affect individual measurements of force.

**Table 1:** Experimental parameters.

Object	Size	Medium	Stage	Laser beam	Value/Error
Silica particle Fig. 4a	$\oslash 1.9\,\mu m$	Water $\mu =0.9\,mPa\cdot s$	—	Gaussian	Trap stiffness: ${k}_{pot}=4.07\pm 0.05\frac{pN}{\mu m}$ ${k}_{map}=4.00\pm 0.02\frac{pN}{\mu m}$
Polystyrene particle Fig. 4b	$\oslash 4.9\,\mu m$	Water $\mu =0.9\,mPa\cdot s$	—	Gaussian	Trap stiffness: ${k}_{pot}=2.22\pm 0.04\,pN/\mu m$ ${k}_{map}=2.18\pm 0.02\,pN/\mu m$
Silica particle Fig. 5a,b	$\oslash 2.2\,\mu m$	Water $\mu =0.9\,mPa\cdot s$	Sine oscillations *f* = 25*Hz*, *Apk* = 5 *μm*	Gaussian *Pest* = 51 *mW*	—
RBC Fig. 5c,d	5.0× $\oslash 8.4\,\mu m$	Plasma $\mu =1.43\,mPa\cdot s$	$v=[50,75,100,125]\,\frac{\mu m}{s}$	Gaussian	Linear fit RMSE: $10.62\cdot {10}^{-14}$ N
‘Blob’ (stretchable) Fig. 5e,f	5.3 × 6.8 *μm*, 5.1 × 7.0 *μm*, 5.0 × 6.8 *μm*, 4.8 × 8.3 *μm*, 4.8 × 8.8 *μm*	Plasma $\mu =1.43\,mPa\cdot s$	$v=[50,75,100,125,150]\,\frac{\mu m}{s}$	Gaussian	Linear fit RMSE: $4.04\cdot {10}^{-14}$ N
Vaterite (birefringent) Fig. 5g,h	$\oslash 1.1\,\mu m$	Ethanol $\mu =1.1\,mPa\cdot s$	${v}_{\perp }=[100,150,200,250,280]\,\frac{\mu m}{s}$; ${v}_{||}=[100,150,200,250,300]\,\frac{\mu m}{s}$	Gaussian	Linear fit RMSE: $\perp :3.88\cdot {10}^{-14}$ N, $\parallel :1.69\cdot {10}^{-14}$ N
Polystyrene particle Fig. 5i,j	$\oslash 3.1\,\mu m$	Water $\mu =0.9\,mPa\cdot s$;	${v}_{\perp }={v}_{\parallel }=[10,20,30,40,50]\,\frac{\mu m}{s}$;	HG10	Linear fit RMSE: $\perp :1.55\cdot {10}^{-14}$ N, $\parallel :1.07\cdot {10}^{-14}$ N

We perform a series of experiments to show that the force calibration with synchronous measurements of force and position, and the mapping method are applicable to arbitrary particles. Theoretical models for the drag force calculations used here are described in detail in the supplementary information (Section 5). All experimental parameters are listed in the Table 1.

**Figure 5:**
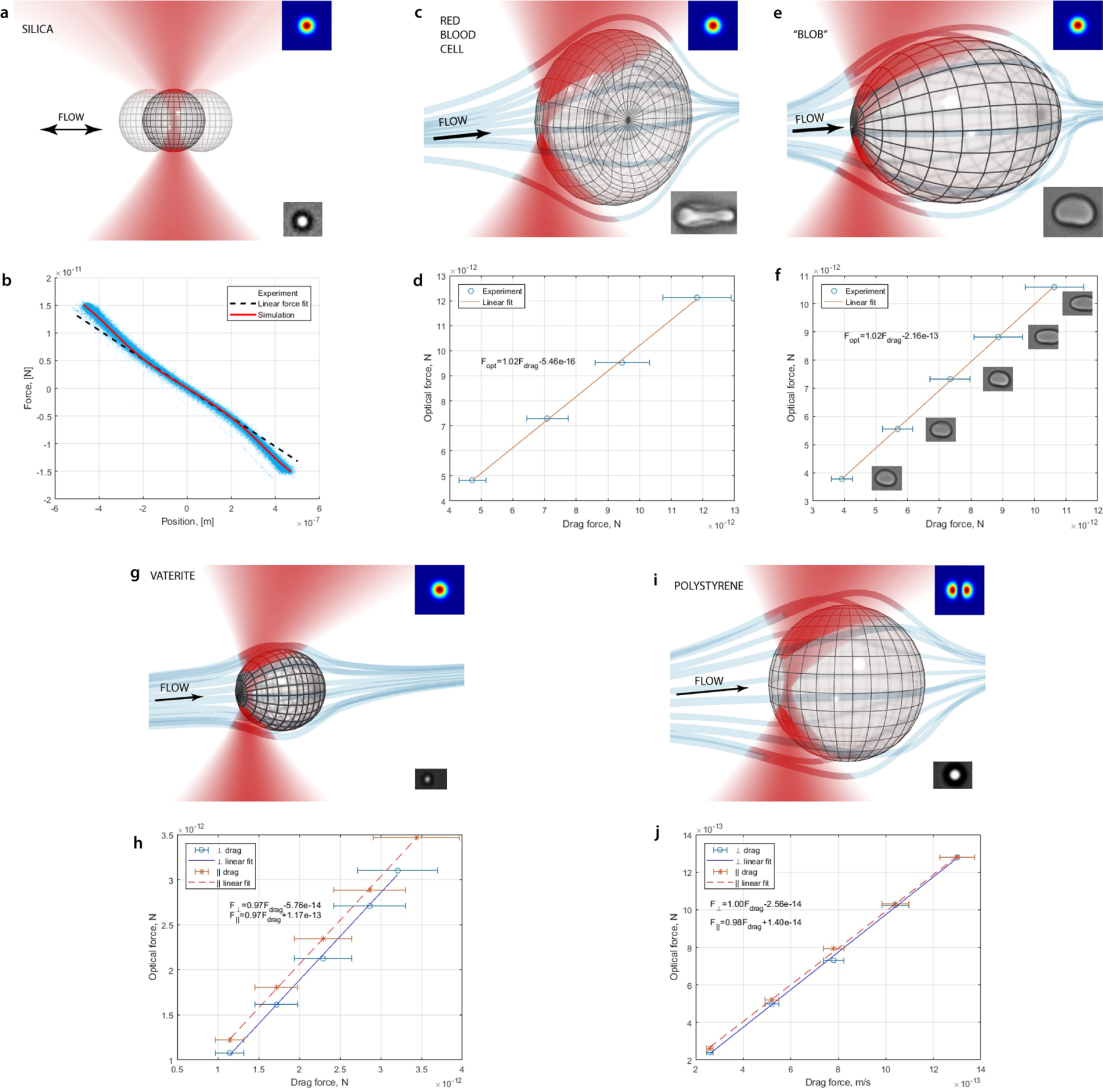
Experimental sketches and results using absolute calibration: (**a**,**b**) Spherical particle in a non-linear region of the force (silica in water, *d* = 2.2 *μm*). (**c**,**d**) RBC in Stokes flow. Fluid drag force acting on the trapped RBC in plasma for different stage velocities. The drag force is that acting on an oblate spheroid of the same size (5.0 × 8.4 *μm*) as the RBC. (**e**,**f**) The blob in Stokes flow. The inset images show the change in shape of the blob as the flow speed changes. Modelled as a prolate spheroid with parameters ranging from 5.3 × 6.8 *μm* to 4.8 × 8.8 *μm*. (**g**,**h**) Birefringent vaterite microsphere in Stokes flow (vaterite in ethanol, *d* = 1.1 *μm*). (**i**,**j**) Spherical particle trapped in an HG_10_ beam in Stokes flow (polystyrene in water, *d* = 3.1 *μm*). Insets in figures (a,c,e,g,i) shows trapped object (bottom) and laser intensity mode (upper).

The first experiment shows the ability of the method to measure the non-linear portion of the force–position curve, i.e., outside the Hookean region of the trap. We trap a spherical silica particle and, by oscillating the stage, we shift the particle into a non-linear region of the trap as in Fig. 5a. The force–position curve and the theoretical calculations are shown in Fig. 5b. The power at the focus (Table 1) was estimated using a measurement of the power before the microscope objective, multiplied by the transmission of the objective. The measurements are in excellent agreement with theoretical predictions.

In the second experiment we trap a human red blood cell (RBC) in plasma using a single beam optical trap. It is hard to theoretically estimate the optical force that is acting on a real non-spherical object in the trap. In order to test our synchronisation method, we apply a fluid drag force to the trapped RBC by moving the stage with a constant velocity. The measured change in the optical force is compared with a theoretical estimation of the drag force. We model the RBC as an oblate spheroid which moves perpendicularly to the symmetry axes as shown in Fig. 5c. Again, our measurements of this RBC, shown in Fig. 5d, are in close agreement with the theoretical calculations. Thus, even a simplified model of the RBC can effectively describe its behaviour.

Next, we show the viability of absolute calibration of an arbitrary particle by measuring the drag force acting on an unknown particle which was found in the sample of RBCs, henceforth referred to as the “blob”, shown in Fig. 5e. Unlike the RBCs, the blob is roughly a prolate spheroid and does not orientate itself in the trap as a RBC does. The measured drag force and the theoretical calculations are shown in Fig. 5f. As the blob’s walls are very soft, we can see a stretching of this structure at high velocities ($v\geqslant 125\,\mu {m}/{s}$). For these cases the blob is stretching out of the region of observation. We measure the size of the blob by fitting an ellipse to the visible part of the blob (which is >90% of the length of the blob). However, even in this situation, the results are in close agreement with the theoretical prediction when the change in the size is taken into account. Thus, we were able to measure the optical force acting on a structure with a variable shape which is impossible to do with other methods.

Next, we show that the absolute calibration is also correct for birefringent particles. Here, we trap a vaterite microsphere^23^, and apply a fluid drag force. Since the vaterite particle aligns with its optic axis in the direction of linear polarisation of the trap^24^, we present results for drag force applied in the direction of polarisation and normal to the direction of polarisation in Fig. 5h. The lines comparing optical to drag forces for the two directions are offset since we assume that the vaterite particle is spherical, and do not account for its small asphericity.

Finally, we demonstrate that the same calibration remains correct if the trapping beam is changed. Here, we use an HG_10_ beam to trap a polystyrene microsphere. Since the focal spot of an HG_10_ consists of two spots in one direction, we show the response for drag force applied both in this direction and normal to it, in Fig. 5j.

## Discussion and Summary

We have introduced a method of absolute force calibration. We have shown how synchronous force and position measurements can be collected experimentally to give an absolute force calibration, and confirmed this experimental arrangement with red blood cells, another biological entity (the blob), and vaterite microspheres. The calibration is also valid if the trapping beam has other than Gaussian distribution and we demonstrated this for an HG_10_ beam. We further show that it is not necessary to take synchronous force and position measurements to obtain an absolute force calibration for a monotonic optical trap, which can be done by mapping the measurements to one another. This absolute calibration through mapping data does not need further assumptions about the probe particle or trapping environment, which is useful when making measurements *in vivo* or in an unknown environment. Notably, mapping, with and without synchronous measurements, does not require the assumption of a Hookean trap for calibration, which expands the range of probe particles, environments and uses of optical tweezers. This implies that these methods of absolute calibration will allow versatile application of optical tweezers in biologically relevant environments where other calibration methods would be difficult or impossible to implement.

The improvements over the equipartition and Boltzmann statistics methods are clearly shown by our simulations and our experimental results. The comparison of the absolute errors in Fig. 2 shows the improvement obtained by not requiring the assumption of a Hookean trap (equipartition and linear fits curves in Fig. 2), and a very large improvement in the determination of the force–position curve with high spatial resolution. In Fig. 4 the improvement in edge statistics compared with the Boltzmann statistics method is clear. Further, in Fig. 5b, we demonstrate measurement of the force–position curve over 4 times the spatial distance available through Brownian motion alone. This additional distance also results in a strongly non-linear force–position curve, where the improved resolution over the Boltzmann statistics method becomes important.

While we have only demonstrated two-dimensional absolute calibration here, the combination of three-dimensional particle tracking^25^ and three-dimensional direct force measurement^8,19^ would allow extension of this work to three dimensions.

In summary, first, force and position measurement with detectors with absolute calibration—PSD for force and CCD camera for position, without using the force–position relationship—can be used to measure optical forces. Such calibration is robust across different particles, trapping mediums, and trapping beams. The particle can be non-spherical, birefringent, or even change shape or size over the course of the measurements. If synchronous force and position measurements can be made, paired force and position data can be obtained at the data acquisition rate of the slower of the two instruments. The force–position relationship can be determined, including outside the Hookean (linear) region of the trap, and does not depend on the measured region of the force–position curve being monotonic. If synchronous force–position measurements are not possible, the force–position relationship can be determined, including outside the Hookean (linear) region of the trap, but only for a region where the force–position curve is monotonic.

Second, the force–position curve can be used to obtain pairs of force and position measurements. Paired force and position data can be obtained at the data acquisition rate of the faster of the two instruments, without needing to obtain synchronous measurements. If force measurements are made with the PSD, and matching positions are determined from them using the force–position curve, position measurements made with the camera (non-synchronous, and at a slower rate) can be used to validate the force measurements. If the particle is non-spherical, the effect of its orientation on the optical force must be considered. Either the particle must remain in the same orientation over the course of the measurements, or the force–position curve must be interpreted in a time-averaged sense^14^. If the particle remains in the same orientation, measurements are not sensitive to the choice of how the centre of the particle is chosen for purposes of tracking). For particles with a high level of symmetry (e.g., cylinders), the choice of centre is obvious. For irregular particle which change orientation due to rotational Brownian motion, one useful strategy will be to choose the particle centre such that the standard deviation of motion of the particle centre is minimised.

## Methods

In the mapping calibration method, assuming that the trap is monotonic, the negative forces should map onto the positive positions and the positive forces should map onto the negative positions. The particle moves around in the trap under Brownian motion with no other applied external forces present in the system. For independent Brownian motion simulations, the positions of the particle and the forces acting on the particle are recorded. They are sorted in ascending order then quantiled into *N* bins of equal sizes. The bins are then mapped to each other such that the *n*th bin of one is mapped to the (*N* − *n*)th bin of the other. This is illustrated further in the supplementary information (Section 1).

The optical system consists of a laser beam (IPG Photonics YLD-5 fiber laser, 1070 nm) which is expanded to fill the back aperture of the objective (water immersion 60×, NA 1.2). A spatial light modulator (SLM) is used for alignment and HG mode generation only and works as a mirror during all other experiments. Collimated light is focused onto the sample chamber made from two cover glasses separated by parafilm and glued at the edges to prevent water motion and evaporation. A dichroic mirror separates the trapping beam from the illumination beam. A high numerical aperture condenser (silicon oil immersion 100×, NA 1.35) collects scattered light which is imaged on the PSD (On-track PSM2-10 with OT-301DL amplifier) using a relay lens.

To get synchronized image-grab and PSD data capture we have developed a program in Labview. Synchronization is achieved by generating a pulse train using a data acquisition card’s (NI-PCIe-6351) built-in timer which is used as a trigger for a series of PSD measurements and as a timer for image grabbing. Our high-speed CMOS camera (Mikrotron MC1362, 1280 × 1024) is capable of acquiring (FrameGrabber NI-PCIe-1433) images at 5kHz framerate with a low level of lost frames (<0.5%). The PSD’s maximum reading rate is 16kHz. Thus, we measure and average three PSD data points at 15kHz per one camera frame.

Camera calibration is performed by tracking the particle’s motion with known displacement. A particle stuck to the slide is moved by the piezo stage (PI P-563.3CD). The ratio of the actual distance to the number of pixels is a camera calibration coefficient. A series of measurements was averaged to suppress the noise in the camera calibration constant *C*_cam_ = 0.170 ± 0.003pxl/*μ*m.

The position of the particle was measured by finding a centroid of the image of a particle

18 $$[X,Y]=[\frac{\int \,xI(x,y)dx}{\int \,I(x,y)dx},\frac{\int \,yI(x,y)dy}{\int I\,(x,y)dy}]$$

where *I*(*x*, *y*) is the value of the pixel at the position [*x*, *y*]. All background pixels are suppressed to 0.

Units of RBCs and plasma were obtained through the Australian Red Cross Blood Service Processing Centre. Informed donors consent was obtained prior to donation for all samples through the Australian Red Cross Blood Service donor questionnaire. This study was approved by the Australian Red Cross Blood Service Human Research Ethics Committee. All experimental methods were performed in accordance with Australian blood authorities guideline and regulations.

Red blood cells used in this paper were sampled from units of leukodepleted packed RBCs in saline-adenine-glucose-mannitol solution. Before measurement, the cells were resuspended in ABO compatible human pooled plasma (i.e., plasma compatible with the ABO blood type of the RBCs) to reproduce a physiological environment.

## Electronic supplementary material

Supplementary Information
