# Supplementary material for: Calibration of force detection for arbitrarily shaped particles in optical tweezers
Source: Sci Rep. 2018 Jul 17;8:10798. doi: 10.1038/s41598-018-28876-y (PMC6050307; doi:10.1038/s41598-018-28876-y)
Supplement: Supplementary file 1 — Supplementary Information [file 41598_2018_28876_MOESM1_ESM.pdf]

Calibration of force detection for arbitrarily shaped particles in optical tweezers

Ann A. M. Bui[1†*], Anatolii V. Kashchuk[1†],Marie Anne Balanant[2,3], Timo A. Nieminen[1], Halina Rubinsztein-Dunlop[1], Alexander B. Stilgoe[1*]

[1]School of Mathematics and Physics, The University of Queensland, St. Lucia QLD 4072, Australia
[2]Australian Red Cross Blood Service, Brisbane QLD 4059, Australia
[3]Science and Engineering Faculty, Queensland University of Technology, Brisbane QLD 4000, Australia
[†]These authors contributed equally
[*]Corresponding authors: ann.bui@uqconnect.edu.au and stilgoe@physics.uq.edu.au

1 Example procedure of mapping method

To demonstrate the mapping procedure, we consider a small scale simulation. A particle undergoes Brownian motion in an optical trap, and no other forces are acting. The simulated optical trap uses the Optical Tweezers Toolbox [1] for the calculation of the optical force. The particle is a 1 μm diameter sphere of refractive index of 1.45, and the surrounding medium is water with refractive index 1.33 and viscosity of 0.91 mPa.s at 298 K. The simulation outputs the force and position of that particle, as shown in Figure 1a, for 10^4 measurements, i.e., that many time steps, in the simulation. These force and position measurements are not synchronised. The force and position data are then sorted into ascending order for the positions, and descending order for the forces, as shown in Figure 1b.

It is typical to have force and position data sets of different sizes, especially if they are not synchronously collected. A PSD typically collects data at higher rates than a CCD, which results in a larger force data set. The data sets can still be broken into the same number of quantiles, which allows the one-to-one correspondence between the quantiles of each data set. Figure 1c shows the sorted force and position data with 15 quantiles marked with circles. The force and position data in Figure 1c is the same data as Figure 1b. Out of those 15 quantiles, the nth quantile of the sorted position measurement becomes the abscissa, and the nth quantile of the sorted force measurements becomes the ordinate, of the force–position curve. Since there were 15 quantiles taken, there are 15 points in the force position curve, as shown in Figure 1d. We also performed an accuracy analysis to investigate the effect of the number of quantiles taken to construct the force–position profile. The term 'bins' and 'quantiles' are used interchangeably to describe the division of the data into quantiles.

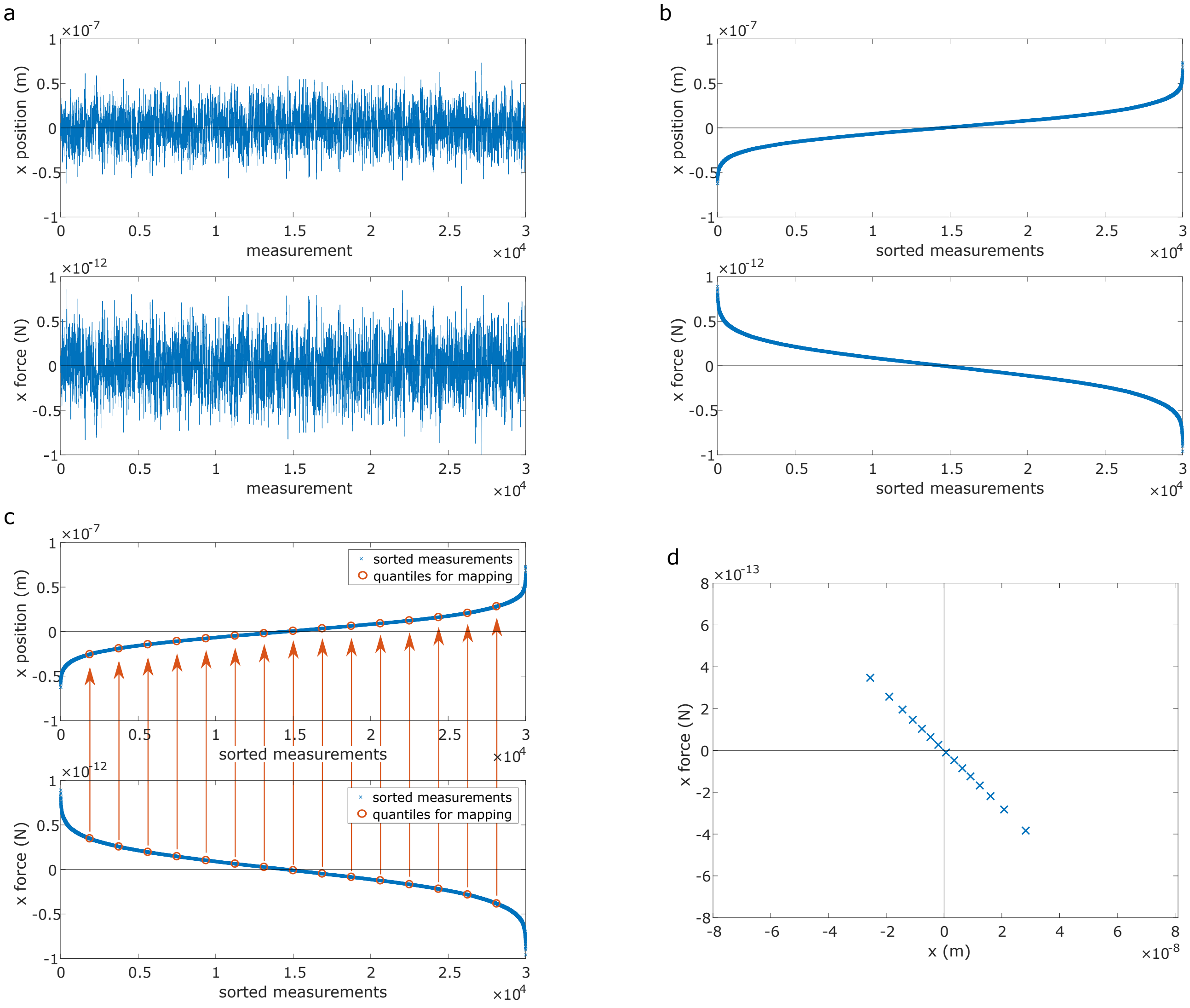

Figure 1: Calibration of simulated optically trapped silica particle in water. There are 10^4 measurements for the force and position of the particle. These force and position data are not synchronised. **a** Raw output of force and position recorded for a trapped particle undergoing Brownian motion. **b** The position data are sorted in ascending order. The force data are sorted in descending order. **c** The force–position curve is constructed by taking the nth sorted position term and the nth sorted force term to create to the nth point in the force–position curve.

1.1 Mapping method with experimental data

We provide an example of the force–position curve calculation from experimental measurements. We use the mapping method and calculations of potential which were used for Figure 4b of the main text. Figure 2 represents raw data for the force and position measurements multiplied by the corresponding calibration constants for the detector and camera.

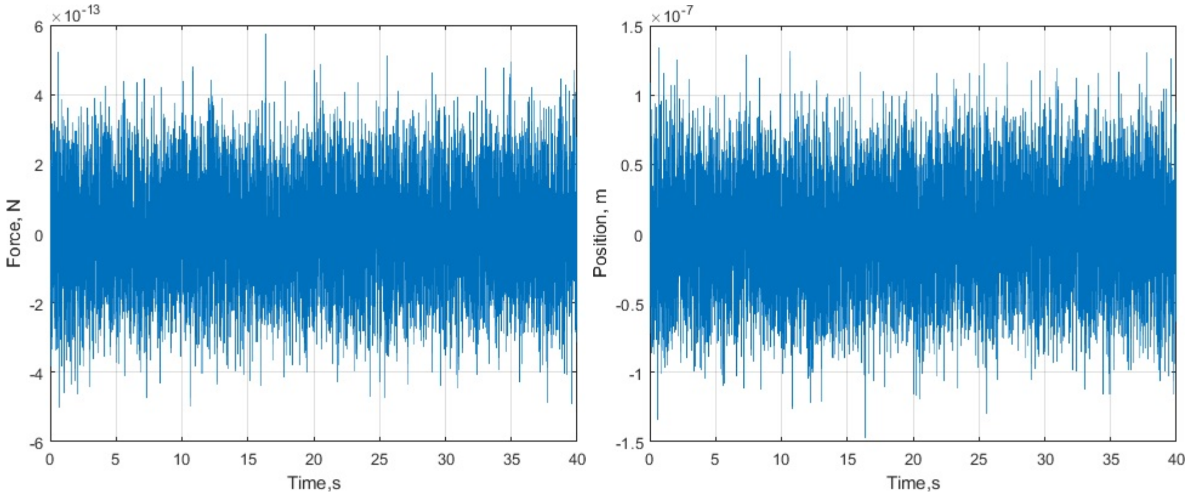

Figure 2: Raw output of force (**left**) and position (**right**) recorded for a trapped particle undergoing Brownian motion.

The next step is to build a histogram of the force and position distribution (Figure 3).

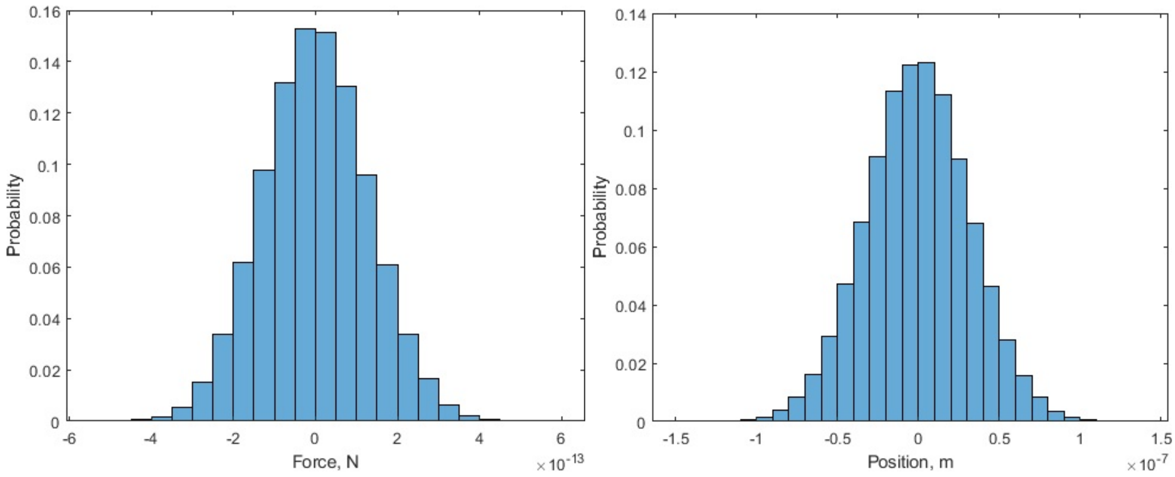

Figure 3: Histogram of the optical force (**left**) and position (**right**) of a trapped particle flipped from left to right.

A quantile–quantile plot (qq-plot) of these distributions directly leads to the force–position curve as shown in Figure 4b of the main text.

The force–position curve can be obtained from calculation of the optical potential. We start with raw data for the position of the particle (Figure 2), bin the data into a histogram and build a potential; the obtained distribution is shown in Figure 4. The derivative of the potential is the force–position curve, as shown in Figure 4b.

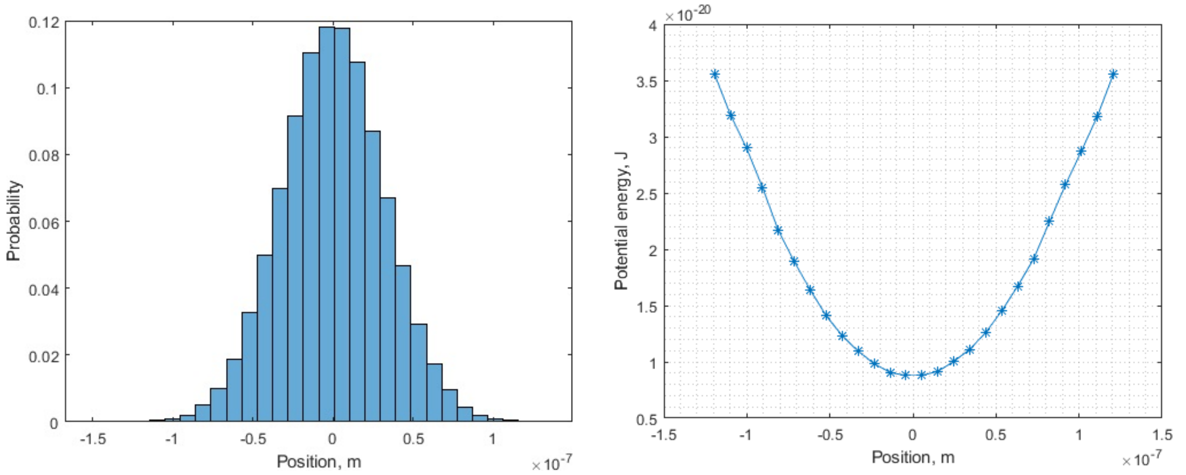

Figure 4: **Left:** position distribution of the trapped particle undergoing Brownian motion. **Right:** potential of the optical trap.

2 Error definition

For the experiment, to define the ‘actual value’ for the force acting on the trapped particle at a particular position, we performed the force calibration on the full length of a run, approximately 200 000 points. The experiment error analysis presented in the main text is performed with 10 000 points, compared to the ‘actual value’ calibration which was done with 200 000 points. For the simulations, we find the axial equilibrium, and calculate the force in the x-direction. The optical force acting on a particle at a particular position can be calculated exactly with the Optical Tweezers Toolbox [1]. More detailed discussion on simulation methods and theory can be found in [2] and [3].

The calibration is performed with all of the collected data. The relative error calculation is performed within a fixed domain, in the centre two-thirds of the space traversed by the trapped particle.

The error is defined to be the average error over the whole calibration, which is

$$\sqrt{\frac{\sum_{x=a}^{b} \delta x \left(f(x) - m(x)\right)^2}{\sum_{x=a}^{b} \delta x \cdot m(x)^2}} \tag{1}$$

where $x = [a, b]$ is a domain over which the error is calculated, $f(x)$ is the value of the force that is calculated with the proposed methods, that is, Boltzmann statistics or sorting, and $m(x)$ is what is accepted to be the actual value for the force at x calculated with the T-matrix method, the same method as used to obtain the data for analysis. This actual curve was taken to be the lateral force, extending outwards from the beam axis at the point of axial equilibrium. For the experimental data, the actual value was taken from a larger data set. The integration of the error is calculated numerically with the trapezoidal method.

3 Noise

Noisy data was defined as:

$$\text{noisy data} = \text{raw data} + \text{noise factor} \cdot \text{std(raw data)}. \tag{2}$$

A noise factor between 0 and 1 was chosen for the simulation.

The shape of the noisy force–position curves is generally preserved when there are more data points, but the error decreases. That is, when more points are taken, all methods are more accurate. In Figure 5, we see that this relative error, collected for analysis of 1 000 000 points is smaller than the relative error presented in the main text where the calculation was performed with 10 000 points.

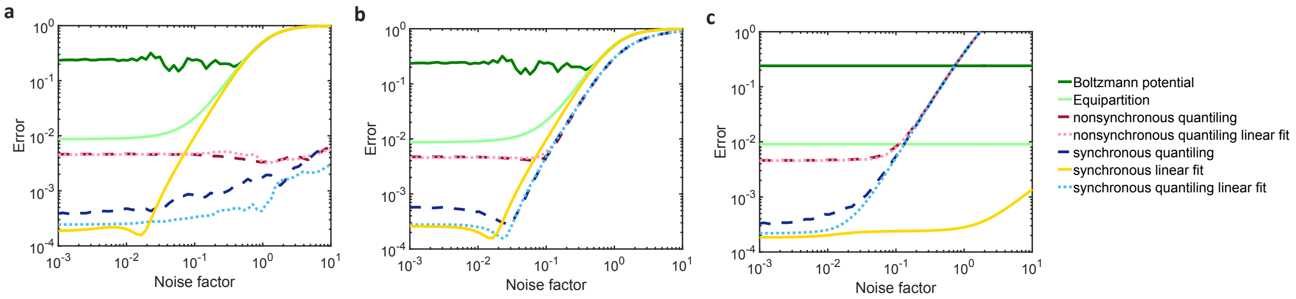

Figure 5: For the methods shown in the main text, we look at the effect of noise for a large number of collected points (1 000 000 points). A noise factor is used as shown in Equation (2). **a** has added noise in equal proportions in the position and force data, **b** has added noise of the noise factor in the position data with fixed noise of 1% in the force data, and **c** has added noise of the noise factor in the force data for a fixed noise of 1% in the position data.

4 Calculation of noise in the experimental system

Figure 6**a** depicts the experimental results showing the behaviour of the relative error as more data points are collected. The relative error considered here is the statistical error. Therefore, we can estimate the relative error from the convergence as a longer data set is used. The "true" force is estimated from the full 200 000 point data set, with 10 000 point segments used to estimate the error. We note that experimental data contains noise, so we considered the performance of the calibration methods with noise. Figure 6**b–d** represents the calibrations with 10^4 data points, and with added Gaussian noise. This noise is a factor of the standard deviation of the data.

From Figure 6**b–d**, we can see that the Boltzmann statistics and equipartition calibration methods are not affected by noise in force measurement. This should not be a surprise since these methods are calculated using only position measurements. The mapping methods begin to be affected by a noise level of about 10%. However, when there is equal noise in force and position, there is less effect on error. This makes sense when one considers that the mapping method orders the measurements, and so long as both sets of data are spread equally, they should still match with each other. Comparing the synchronous and nonsynchronous calibrations, the synchronous method becomes as accurate as the nonsynchronous methods with noise. This is true for calibration with and without linear fitting, even though the synchronous measurements have less error with minimal noise. The linear-fitted synchronous measurements respond well to added noise in force measurements, but behave like the mapping method with noise when there is noise in the position data. In general, for noise of less than 10% of the standard deviation of data, any of these methods should respond well. Comparing the relative noise of the experiment with Figure 6**a** at 10^4 data points, to the relative noise of Figure 6**b–d**, we can conclude that there is roughly 10% noise in the experimental data. This noise level corresponds to position resolution of a fiftieth of a pixel, at roughly 300 nm/pixel.

5 Drag force calculations

To estimate the drag force on the red blood cell (RBC) we model the RBC as an oblate spheroid which is (due to reorientation in the trap) moving perpendicularly to the axis of symmetry. In this case, the

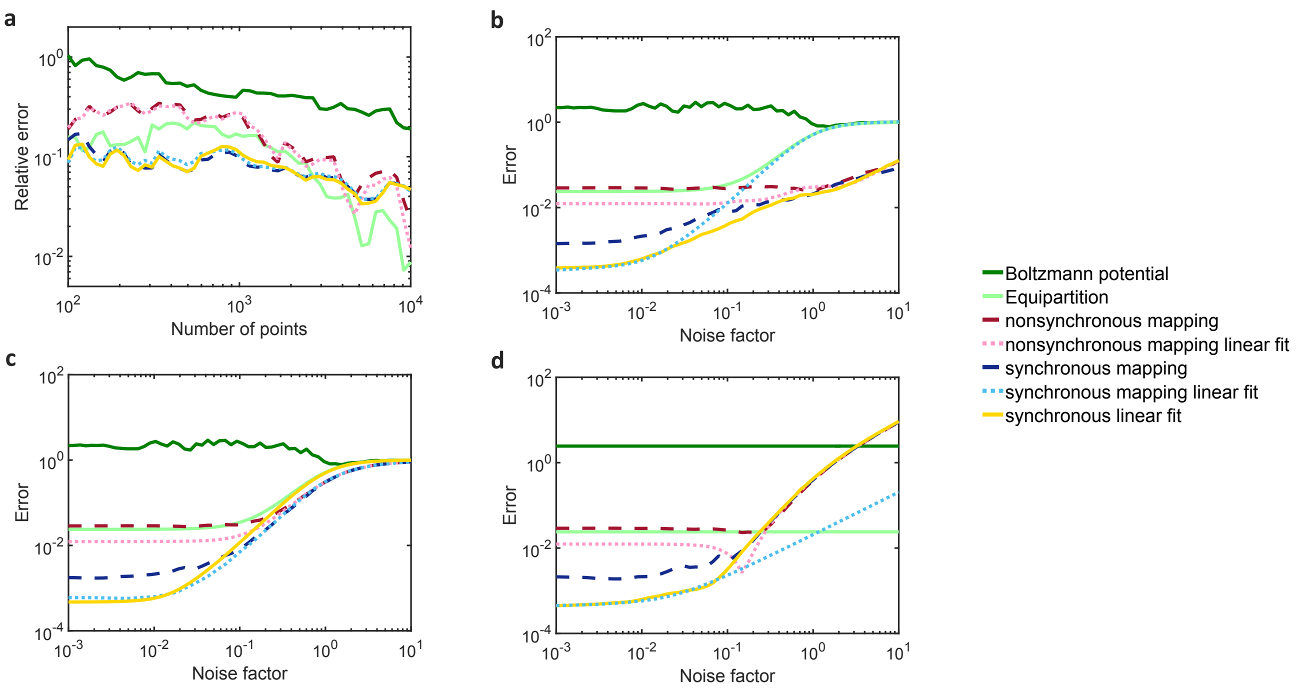

Figure 6: **a** shows the effect of the number of force and position data points collected on the error. **b–d** show the error associated with noise for 10 000 points, i.e., the number of data points used in the main text. **b** has added noise in equal proportions in the position and force data, **c** has added noise of the noise factor in the position data with fixed noise of 1% in the force data, and **d** has added noise of the noise factor in the force data for a fixed noise of 1% in the position data.

drag force can be calculated as [4]

$$F = 6\pi\mu VR \tag{3}$$

where R is the radius of an equivalent sphere normalized by the symmetry semi-axis lengths of the spheroid:

$$R_{\parallel} = \frac{4}{3}\frac{q}{\alpha_0(1-2q^2)+2q^2}, \tag{4}$$

$$R_{\perp} = \frac{8}{3}\frac{q}{\alpha_0(3-2q^2)+2q^2}. \tag{5}$$

In these equations, the subscripts indicate fluid flow parallel ($\|$) and perpendicular ($\perp$) to the symmetry axis, q is the ratio of the spheroid axes ($q < 1$ for oblate spheroid and $q > 1$ for prolate spheroid), and α_0 is defined as:

$$\alpha_0(q<1) = -\frac{q^2}{1-q^2} + \frac{q}{(1-q^2)^{\frac{3}{2}}}\tan^{-1}\frac{\sqrt{1-q^2}}{q}, \tag{6}$$

$$\alpha_0(q>1) = -\frac{q^2}{q^2-1} - \frac{q}{2(q^2-1)^{\frac{3}{2}}}\ln\frac{q+\sqrt{q^2-1}}{q-\sqrt{q^2-1}}. \tag{7}$$

For the estimation of the drag force acting on the RBC we use an oblate spheroid model with a flow perpendicular to the symmetry axis (equations 5 and 6). The blob is assumed to be a prolate spheroid with a flow parallel to the symmetry axes (equations 4 and 7). For both RBC and blob drag force calculations, the viscosity of the plasma is $\mu = 1.43 \pm 0.08$ cP [5].

References

[1] Nieminen, T. A. et al. Optical tweezers computational toolbox. *Journal of Optics A: Pure and Applied Optics* 9, S196-S203 (2007).

[2] Bui, A. A. M. et al. Theory and practice of simulation of optical tweezers. *Journal of Quantitative Spectroscopy and Radiative Transfer* 195, 66-75(2017).

[3] Nieminen, T. A. et al. Optical tweezers: theory and modelling. *Journal of Quantitative Spectroscopy and Radiative Transfer* 146, 59-80 (2014).

[4] Weinheimer, A. J. Application of the Stokes drag on spheroids to the drag on disks and cylinders *Journal of the Atmospheric Sciences* V44, 2674-2676 (1987).

[5] Maugeri, D. et al., Alterations of blood and plasma viscosity and erythrocyte filtration in senile osteoporosis. *Archives of Gerontology and Geriatrics*, V19, 75-82 (1994).
